# Reliability of CD44, CD24, and ALDH1A1 immunohistochemical staining: Pathologist assessment compared to quantitative image analysis

**DOI:** 10.3389/fmed.2022.1040061

**Published:** 2022-12-14

**Authors:** Lusine Yaghjyan, Yujing J. Heng, Gabrielle M. Baker, Vanessa Bret-Mounet, Divya Murthy, Matt B. Mahoney, Yi Mu, Bernard Rosner, Rulla M. Tamimi

**Affiliations:** ^1^Department of Epidemiology, College of Public Health and Health Professions and College of Medicine, University of Florida, Gainesville, FL, United States; ^2^Department of Pathology, Beth Israel Deaconess Medical Center and Harvard Medical School, Boston, MA, United States; ^3^Channing Division of Network Medicine, Department of Medicine, Brigham and Women’s Hospital and Harvard Medical School, Boston, MA, United States; ^4^Department of Population Health Sciences, Weill Cornell Medicine, New York, NY, United States

**Keywords:** stem cell markers, benign breast disease (BBD), automated image analysis, intra-class correlation (ICC), immunohistochemistry

## Abstract

**Background:**

The data on the expression of stem cell markers CD44, CD24, and ALDH1A1 in the breast tissue of cancer-free women is very limited and no previous studies have explored the agreement between pathologist and computational assessments of these markers. We compared the immunohistochemical (IHC) expression assessment for CD44, CD24, and ALDH1A1 by an expert pathologist with the automated image analysis results and assessed the homogeneity of the markers across multiple cores pertaining to each woman.

**Methods:**

We included 81 cancer-free women (399 cores) with biopsy-confirmed benign breast disease in the Nurses’ Health Study (NHS) and NHSII cohorts. IHC was conducted with commercial antibodies [CD44 (Dako, Santa Clara, CA, USA) 1:25 dilution; CD24 (Invitrogen, Waltham, MA, USA) 1:200 dilution and ALDH1A1 (Abcam, Cambridge, United Kingdom) 1:300 dilution]. For each core, the percent positivity was quantified by the pathologist and Definiens Tissue Studio. Correlations between pathologist and computational scores were evaluated with Spearman correlation (for categorical positivity: 0, >0–<1, 1–10, >10–50, and >50%) and sensitivity/specificity (for binary positivity defined with 1 and 10% cut-offs), using the pathologist scores as the gold standard. Expression homogeneity was examined with intra-class correlation (ICC). Analyses were stratified by core [normal terminal duct-lobular units (TDLUs), benign lesions] and tissue type (epithelium, stroma).

**Results:**

Spearman correlation between pathologist and Definiens ranged between 0.40–0.64 for stroma and 0.66–0.68 for epithelium in normal TDLUs cores and between 0.24–0.60 for stroma and 0.61–0.64 for epithelium in benign lesions. For stroma, sensitivity and specificity ranged between 0.92–0.95 and 0.24–0.60, respectively, with 1% cut-off and between 0.43–0.88 and 0.73–0.85, respectively, with 10% cut-off. For epithelium, 10% cut-off resulted in better estimates for both sensitivity and specificity. ICC between the cores was strongest for CD44 for both stroma and epithelium in normal TDLUs cores and benign lesions (range 0.74–0.80). ICC for CD24 and ALDH1A ranged between 0.42–0.63 and 0.44–0.55, respectively.

**Conclusion:**

Our findings show that computational assessments for CD44, CD24, and ALDH1A1 exhibit variable correlations with manual assessment. These findings support the use of computational platforms for IHC evaluation of stem cell markers in large-scale epidemiologic studies. Pilot studies maybe also needed to determine appropriate cut-offs for defining staining positivity.

## 1 Introduction

Breast tissue undergoes significant structural changes throughout the woman’s life (1). The tissue architecture is maintained by a population of stem cells with self-renewal capacity, which are essential for tissue repair and remodeling (2). However, potentially limitless proliferative and self-renewal capacity, and high susceptibility to various endogenous and exogenous mutagenic insults increase the chances of their tumorigenic transformation (1, 3). Further, in the mammary gland, stem cells are the only cell subpopulation that can accumulate all the oncogenic alterations (1). Recently, the role of stem cells in breast cancer development, progression, and treatment has been recognized as a high priority for translational breast cancer research (4).

Well-characterized stem cell markers CD44 and CD24 have been linked to younger age at diagnosis, higher odds of unfavorable tumor characteristics, including triple-negative status and distant metastasis (5–8). Another stem cell marker, aldehyde dehydrogenase family 1 member A1 (ALDH1A1), is correlated with poor prognosis and chemotherapy resistant breast cancer (7, 9–13). However, the data on the expression of stem cell markers in the breast tissue of cancer-free women for utilization in studies of associations with breast cancer risk or risk prediction is very limited.

Tissue microarrays (TMAs) coupled with digital pathology image analysis software allow for more precise quantification of tissue biomarker expression across a large number of samples, thus greatly enhancing the rigor of the tissue data for epidemiologic investigations (14, 15). Previous studies of selected markers in tumor breast tissue have demonstrated high agreement between pathologist reading and computerized assessment of expression for ER, PR, HER-2, and Ki67 in breast tumor tissue (14, 16, 17). However, it is unclear whether this agreement extends to stem cell markers biomarkers in non-malignant breast tissue Understanding the extent to which automated platforms concur with pathologist assessments can help in determining the appropriate analytic method to evaluate immunoreactivity, particularly in analyses using archival formalin-fixed paraffin-embedded (FFPE) tissues. To our knowledge, no previous studies have explored the agreement between pathologist manual read and computer-derived expression of stem cell markers CD44, CD24, and ALDH1A1, which may have potential implications for large-scale epidemiological studies focusing on stem cell hypothesis of breast carcinogenesis.

To fill these knowledge gaps, in this study we aimed to (1) describe the expression of CD44, CD24, and ALDH1A1 in non-malignant breast tissue of cancer-free women; (2) assess the homogeneity of their expression across multiple cores pertaining to each woman; and (3) compare the immunohistochemical expression assessment for CD44, CD24, and ALDH1A1 by an expert pathologist with the automated image analysis results.

## 2 Materials and methods

### 2.1 Study population

Our analysis included cancer-free women with biopsy-confirmed benign breast disease (BBD) in the Nurses’ Health Study (NHS) and Nurses’ Health Study II (NHSII) cohorts who were previously included in a nested case-control study of breast cancer (18, 19). These prospective cohorts followed registered nurses in the United States who were 30–55 years (NHS) or 25–42 years old (NHSII) at enrollment. After administration of the initial questionnaire, the information on breast cancer risk factors and any diagnoses of cancer (subsequently confirmed *via* medical record) or other diseases (including BBD) was updated through biennial questionnaires (20, 21). Details of this nested case-control study and the BBD assessment have been previously described (18, 19).

Early NHS questionnaires (1976, 1978, and 1980) asked whether the participant had ever been diagnosed with “fibrocystic disease” or “other BBD” and whether she had been hospitalized in relation to this diagnosis. Beginning in 1982, the NHS questionnaires specifically asked about a history of biopsy-confirmed BBD (fibrocystic disease or other BBD). The initial 1989 NHS II questionnaire and all subsequent biennial questionnaires also asked participants to report any diagnosis of BBD and to indicate whether it was confirmed by biopsy or aspiration.

Cases were defined as women with biopsy-confirmed BBD who reported a diagnosis of breast cancer during 1976–1998 for the NHS and 1989–1999 for the NHSII following their BBD diagnosis. Using incidence density sampling, four women with biopsy-confirmed BBD who were free of breast cancer at the time of the matching case’s diagnosis (controls) were matched to the respective case on year of birth and year of benign breast biopsy (22). We attempted to obtain BBD pathology records and archived biopsy specimens for all cases and controls from their hospital pathology departments; our ability to obtain biopsy blocks did not significantly differ by case and control status. Women with and without BBD samples were similar with respect to the distribution of breast cancer risk factors (23). Our study included 81 women with 399 corresponding tissue cores.

The study protocol was approved by the institutional review boards of the Brigham and Women’s Hospital and Harvard T.H. Chan School of Public Health and those of participating registries as required. Consent was obtained or implied by return of questionnaires.

### 2.2 Benign breast biopsy confirmation

Hematoxylin and eosin (H&E) breast tissue slides were retrieved for biopsy-confirmed BBD patients who gave permission to review their biopsy records. The slides were independently reviewed by one of three pathologists in a blinded fashion, i.e., the evaluating pathologists were blinded to type of BBD noted on the original diagnosis (24, 25). Any slide identified as having either questionable atypia or atypia was jointly reviewed by two pathologists. For each set of slides, a detailed work sheet was completed and the benign breast biopsy was classified according to the categories of Page et al. (26) as non-proliferative, proliferative without atypia, or atypical hyperplasia (ductal or lobular hyperplasia) (18).

### 2.3 Tissue microarray (TMA) construction of BBD samples

After centralized review of hematoxylin and eosin (H&E) stained sections, we collected archived FFPE benign breast biopsy blocks for participants. H&E sections of the corresponding FFPE tissue blocks were re-reviewed by a single pathologist to identify areas of benign proliferative lesions and normal terminal duct-lobular units (TDLUs), and to circle the areas from which the cores for the TMAs would be taken. Normal TDLUs were regions of histopathologically normal tissue that may or may not be adjacent to benign lesions (e.g., atypical ductal hyperplasia, usual ductal hyperplasia) (18). TMAs were constructed at the Dana Farber/Harvard Cancer Center (DF/HCC) Tissue Microarray Core Facility by obtaining 0.6-mm cores from benign lesions and TDLUs. For each woman, up to 3 cores of normal TDLU were included in the TMA blocks. We previously evaluated our TMA construction methods and confirmed a high success rate (76%) of capturing normal TDLUs in these TMA blocks (27).

### 2.4 Immunohistochemistry (IHC) for stem cell markers

In this study, we included one TMA that corresponded to 399 cores from 81 women. The expression of the stem cell markers was evaluated by automated IHC technique that allows the quantification of markers’ expression levels and localization of the target signal to specific cells/structures. For each of the three markers one 5-μm paraffin section was cut from a single TMA block and then stained with antibodies for CD44, CD24, and ALDH1A1 at the University of Florida Pathology Core Lab on DAKO AutostainerPlus according to the previously standardized protocol with commercial antibodies [DAKO AutostainerPlus, CD44 (Dako, Santa Clara, CA, USA) 1:25 dilution; CD24 (Invitrogen, Waltham, MA, USA) 1:200 dilution and ALDH1A1 (Abcam, Cambridge, United Kingdom) 1:300 dilution]. Details of this protocol have been described previously (28, 29). Briefly, slides were de-paraffinized with xylene and re-hydrated through decreasing concentrations of ethanol to water, including an intermediate step to quench endogenous peroxidase activity (3% hydrogen peroxide in methanol) and transferred to 1X TBS-T (Tris-buffered saline-Tween). For heat-induced antigen retrieval, sections were heated in a steamer while submerged in Citra (Biogenex, Fremont, CA, USA) or Trilogy (Cell Marque, Rocklin, CA, USA) for 30 min. Next, slides were (1) rinsed in 1XTBS-T and incubated with a universal protein blocker Sniper (Biocare Medical, Walnut Creek, CA, USA) for 10 (for CD44 and ALDH1A1) or 15 min (for CD24); (2) rinsed in 1XTBS-T and co-incubated in primary antibody ALDH1A1, CD24, or CD44 for 1 h; and (3) rinsed in 1XTBS-T followed by application of conjugated secondary antibody [Mach 2 goat anti-rabbit horse (or mouse) radish peroxidase-conjugated, Biocare Medical, Walnut Creek, CA, USA] for 30 min. Detection of antibodies was achieved by incubating slides in 3′3′ diaminobenzidine (Vector Laboratories Inc., Burlingame, CA, USA) for 4 min. Slides were counterstained with hematoxylin (Biocare Medical, Walnut Creek, CA, USA) 1:10 for 3 min and mounted with Cytoseal XYL (Richard-Allen Scientific, Kalamazoo, MI, USA). The laboratory implemented standard quality control procedures.

### 2.5 Image analysis

Immunoreactivity was manually assessed by an expert breast pathologist (GB) in the Department of Pathology at Beth Israel Deaconess Medical Center. For manual read, the staining extent was quantified as percent of the cells stained positive for each markers out of the total area of the core (0, >0–<1, 1–10, >10–50, and >50%), consistent with the assessment of other tissue markers (30–32) and distribution in our study sample. The assessment was performed separately in epithelium and stroma.

**FIGURE 1:**
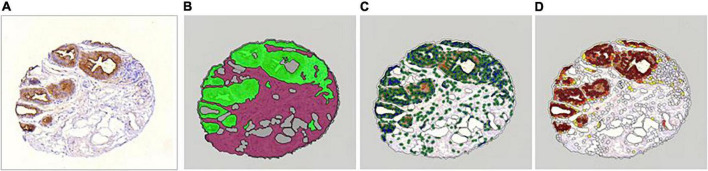
Definiens workflow (This example core was manually read as >50% in epithelium and 1–10% in stroma). **(A)** Raw core. **(B)** Automated tissue segmentation (epithelium in green, stroma in magenta). **(C)** Automated cell detection using nuclei (detected nuclei are circled in green). **(D)** Automated cell scoring (cells with brown chromogen are highlighted with yellow, orange, and brown overlay to reflect chromogen intensity).

Immunohistochemical was also quantified using an automated image analysis system, Definiens Tissue Studio software (Munich, Germany) which quantifies morphology and tissue marker expression within the context of tissue architecture (Figure 1). For each core, the extent of the each marker expression was assessed on a continuous scale as% of cells that stain positively (across all intensities) for a specific marker out of the total cell count, separately for epithelium and stroma. Briefly, TMA slides were digitalized at 20 × into whole slide images using the Pannoramic Scan 150P (3DHistech, Budapest, Hungary). For each marker, the images were imported into Definiens and an experienced operator randomly selected a representative TMA as the training TMA. On the training TMA, the operator randomly selected 12 training cores that were assessed as >0–<1 (*n* = 3), 1–10 (*n* = 3), >10–50 (*n* = 3), and >50% (*n* = 3) by the pathologist to optimize a Definiens’ algorithm for automated IHC assessment. A maximum of 12 cores was allowed by Definiens for algorithm training. The minimum positive IHC staining threshold in Definiens was set using the pathologist’s manual reads as reference. The optimized Definiens algorithm segmented each tissue core into epithelium, stroma, fat, and background, detected the number of cells, and quantitated the IHC stains. Since the cores on these TMAs were specifically constructed to contain TDLUs and benign lesions, our results will be specific to these regions of interest.

### 2.6 Statistical analysis

For analysis, the continuous data from Definiens assessment was categorized as 0, >0–<1, 1–10, >10–50, and >50% consistent with pathologist assessment. Next, as there is no standardized criteria for defining binary staining positivity for CD44, CD24, and ALDHA1, especially in non-cancerous breast tissue (33) and as previous studies in breast tumors used varying criteria [1, 5, 10, or 25% + staining (33–38)], we also used 1 and 10% cut-offs (based on the distribution in our study sample). Distributions of stem cell markers were presented as numbers and percentages, by assessment method, core type (normal tissue or benign lesions) and tissue type (epithelium, stroma).

Intra-class correlation coefficient and 95% Confidence Interval (95% CI) were used to assess homogeneity of stem cell markers’ expression across available cores for a woman, separately for normal tissue and benign lesions as well as by the tissue type (epithelium, stroma, combined epithelium, and stroma).

We used Spearman’s rank-ordered correlation coefficients to examine correlation between pathologist and Definiens expression readings by type of core (normal TDLUs vs. lesions) and tissue (stroma vs. epithelium). For binary positivity variables with 1 and 10% cut-offs, we calculated sensitivity and specificity, while treating pathologist assessment as a gold standard. In additional analyses, these correlations as well as sensitivity and specificity were examined while accounting for correlation across available cores for a woman.

All analyses were limited to cores with at least 50 cells of specific tissue type (epithelium or stroma) from Definiens readings, consistent with previously used approaches (16). The analyses were performed using SAS software (version 9.4, SAS Institute, Cary, NC, USA).

## 3 Results

**FIGURE 2:**
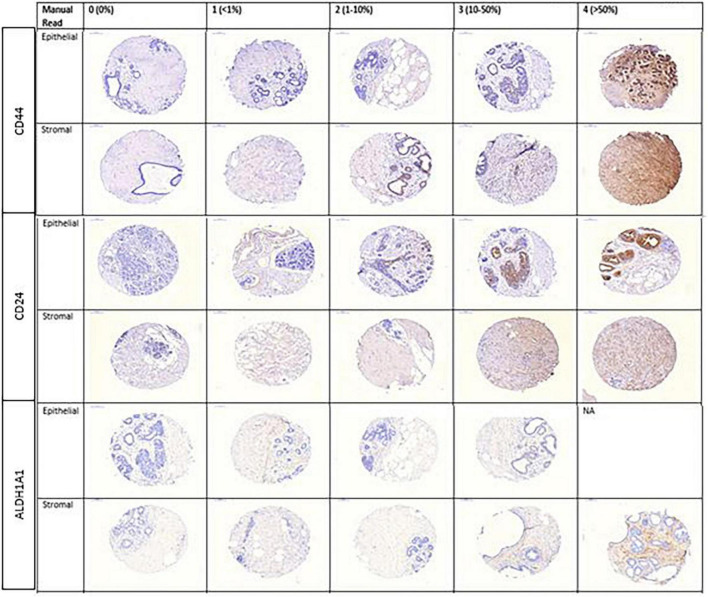
Representative images of CD44, CD24, and ALDH1A1 markers by pathologist-assigned category.

Representative images of IHC stained cores for each of the markers by pathologist-assigned category are shown in Figure 2. Among 399 cores in our study, 219 (54.9%) were normal TDLU tissue cores and 180 represented various benign lesions (51 atypical ductal hyperplasia, 18 atypical lobular hyperplasia, 33 apocrine metaplasia, 12 non-apocrine cysts, and 66 usual ductal hyperplasia). Pathologist readings were available for 325 cores (81%) for stroma and 192 cores (48%) for epithelium for CD44; 322 cores (81%) for stroma and 199 cores (50%) for epithelium for CD24, and 344 cores (86%) for stroma and 200 cores (50%) for epithelium for ALDH1A1. Definiens assessment results were available for 330 cores (82%) for stroma and 318 (66%) cores for epithelium for CD44; 338 cores (83%) for stroma and 329 (68%) cores for epithelium for CD24; and 334 cores (74%) for stroma and 325 (67%) cores for epithelium for ALDH1A1. Of these readings, 4 cores were excluded due to low cellularity (cell count < 50) for stroma and 56 for epithelium for CD44; 8 cores for stroma and 56 for epithelium for CD24; and 39 cores for stroma and 56 for epithelium for ALDH1A1.

### 3.1 Expression of CD44, CD24, and ALDH1A1 in benign biopsy samples and heterogeneity across the cores

Distribution of continuous CD44, CD24, and ALDH1A1 expression in stroma and epithelium of normal cores and lesions from automated assessment is presented in Table 1. Distribution of categorical expression results from pathologist and Definiens assessment is presented in Table 2. The average marker expression in stroma and epithelium appeared to be greater in lesions as compared to normal cores for CD44 (*p* < 0.01 and 0.01, respectively) and greater for CD24 in epithelium (*p* = 0.01). The expression of ALDH1A1 in stroma and epithelium were similar in normal TDLUs and benign lesions. For all three markers, the average expression was greater in epithelium as compared to stroma across core types (*p* < 0.001 for all markers).

**TABLE 1:** Distribution of continuous marker staining (% positivity) assessed with Definiens using a single tissue microarray from Nurses’ Health Study I and Nurses’ Health Study II.

	All cores			Normal TDLUs cores			Benign lesion cores			
Tissue marker/Tissue type	*N*	Mean (STD)	Range	*N*	Mean (STD)	Range	*N*	Mean (STD)	Range	*P* for differences in Normal vs. Lesions
**CD44**										
Stroma	326	15.6 (22.5)	0–97.9	175	13.4 (20.4)	0–91.1	151	18.2 (24.4)	0–97.9	<0.01
Epithelium	262	43.4 (32.9)	0–100	138	39.7 (31.9)	0–99.3	124	47.6 (33.5)	0.4–100	0.01
*P* for difference in Stroma vs. Epithelium		<0.001			<0.001			<0.001		
**CD24**										
Stroma	330	10.5 (17.9)	0–85.6	183	9.7 (18.1)	0–84.9	147	11.5 (17.6)	0–85.6	0.12
Epithelium	273	32.6 (28.0)	0.5–99.6	148	28.0 (25.3)	0.5–97.8	125	38.1 (30.1)	1.0–99.6	<0.01
*P* for difference in Stroma vs. Epithelium		<0.001			<0.001			<0.001		
**ALDH1A1**										
Stroma	295	11.6 (15.7)	0–89.0	163	11.8 (17.1)	0–89.0	132	11.4 (13.8)	0–63.9	0.49
Epithelium	269	26.0 (18.5)	0.5–78.9	147	26.2 (18.9)	0.5–68.3	122	25.7 (18.1)	0.6–78.9	0.76
*P* for difference in Stroma vs. Epithelium		<0.001			<0.001			<0.001		

STD, standard deviation; TDLU, terminal duct lobular unit.

**TABLE 2:** Distribution of CD44, CD24, and ALDH1A1 expression assessments from pathologist and Definiens readings [*N* of cores (%)].

	Pathologist			Definiens		
Tissue marker/Tissue type	All cores	Normal TDLUs	Benign lesions	All cores	Normal TDLUs	Benign lesions
**CD44**						
**Categorical**						
**Stroma**						
0	112 (34.46)	55 (31.43)	57 (38.00)	62 (19.02)	35 (20.00)	27 (17.88)
> 0 <1	61 (18.77)	38 (21.71)	23 (15.33)	40 (12.27)	24 (13.71)	16 (10.60)
≥1–≤10	66 (20.31)	31 (17.71)	35 (23.33)	100 (30.67)	57 (32.57)	43 (28.48)
>10–≤50	55 (16.92)	33 (18.86)	22 (14.67)	91 (27.91)	46 (26.29)	45 (29.80)
>50	31 (9.54)	18 (10.29)	13 (8.67)	33 (10.12)	13 (7.43)	20 (13.25)
**Epithelium**						
0	30 (15.63)	16 (15.84)	14 (15.38)	5 (1.91)	5 (3.62)	0
> 0 <1	6 (3.13)	4 (3.96)	2 (2.20)	4 (1.53)	1 (0.72)	3 (2.42)
≥1–≤10	34 (17.71)	20 (19.80)	14 (15.38)	45 (17.18)	24 (17.39)	21 (16.94)
>10–≤50	80 (41.67)	40 (39.60)	40 (43.96)	98 (37.40)	57 (41.30)	41 (33.06)
>50	42 (21.88)	21 (20.79)	21 (23.08)	110 (41.98)	51 (36.96)	59 (47.58)
**Binary, with 1% cut-off**						
**Stroma**						
<1	173 (53.23)	93 (53.14)	80 (53.33)	102 (31.29)	59 (33.71)	43 (28.48)
≥1	152 (46.77)	82 (46.86)	70 (46.67)	224 (68.71)	116 (66.29)	108 (71.52)
Epithelium						
<1	36 (18.75)	20 (19.80)	16 (17.58)	9 (3.44)	6 (4.35)	3 (2.42)
≥1	156 (81.25)	81 (80.20)	75 (82.42)	253 (96.56)	132 (95.65)	121 (97.58)
**Binary, with 10% cut-off**						
**Stroma**						
≤10	239 (73.54)	124 (70.86)	115 (76.67)	202 (61.96)	116 (66.29)	86 (56.95)
>10	86 (26.46)	51 (29.14)	35 (23.33)	124 (38.04)	59 (33.71)	65 (43.05)
**Epithelium**						
≤10	70 (36.46)	40 (39.60)	30 (32.97)	54 (20.61)	30 (21.74)	24 (19.35)
>10	122 (63.54)	61 (60.40)	61 (67.03)	208 (79.39)	108 (78.26)	100 (80.65)
**CD24**						
**Categorical**						
**Stroma**						
0	188 (58.39)	105 (59.32)	83 (57.24)	40 (12.12)	33 (18.03)	7 (4.76)
> 0 <1	74 (22.98)	34 (19.21)	40 (27.59)	59 (17.88)	36 (19.67)	23 (15.65)
≥1–≤10	43 (13.35)	30 (16.95)	13 (8.97)	145 (43.94)	72 (39.34)	73 (49.66)
> 10–≤50	13 (4.04)	7 (3.95)	6 (4.14)	66 (20.00)	30 (16.39)	36 (24.49)
>50	4 (1.24)	1 (0.56)	3 (2.07)	20 (6.06)	12 (6.56)	8 (5.44)
**Epithelium**						
0	56 (28.14)	30 (28.30)	26 (27.96)	0	0	0
> 0 <1	41 (20.60)	24 (22.64)	17 (18.28)	3 (1.10)	2 (1.35)	1 (0.80)
≥1–≤10	34 (17.09)	19 (17.92)	15 (16.13)	71 (26.01)	43 (29.05)	28 (22.40)
>10–≤50	42 (21.11)	24 (22.64)	18 (19.35)	123 (45.05)	71 (47.97)	52 (41.60)
>50	26 (13.07)	9 (8.49)	17 (18.28)	76 (27.84)	32 (21.62)	44 (35.20)
**Binary, with 1% cut-off**						
**Stroma**						
<1	262 (81.37)	139 (78.53)	123 (84.83)	99 (30.00)	69 (37.70)	30 (20.41)
≥1	60 (18.63)	38 (21.47)	22 (15.17)	231 (70.00)	114 (62.30)	117 (79.59)
**Epithelium**						
<1	97 (48.74)	54 (50.94)	43 (46.24)	3 (1.10)	2 (1.35)	1 (0.80)
≥1	102 (51.26)	52 (49.06)	50 (53.76)	270 (98.90)	146 (98.65)	124 (99.20)
**Binary, with 10% cut-off**						
**Stroma**						
≤10	305 (94.72)	169 (95.48)	136 (93.79)	244 (73.94)	141 (77.05)	103 (70.07)
>10	17 (5.28)	8 (4.52)	9 (6.21)	86 (26.06)	42 (22.95)	44 (29.93)
**Epithelium**						
≤10	131 (65.83)	73 (68.87)	58 (62.37)	74 (27.11)	45 (30.41)	29 (23.20)
>10	68 (34.17)	33 (31.13)	35 (37.63)	199 (72.89)	103 (69.59)	96 (76.80)
**ALDH1A1**						
**Categorical**						
**Stroma**						
0	94 (27.33)	56 (29.47)	38 (24.68)	32 (10.85)	20 (12.27)	12 (9.09)
> 0 <1	73 (21.220)	42 (22.11)	31 (20.13)	19 (6.44)	10 (6.13)	9 (6.82)
≥1–≤10	80 (23.26)	37 (19.47)	43 (27.92)	143 (48.47)	82 (50.31)	61 (46.21)
>10–≤50	64 (18.60)	34 (17.89)	30 (19.48)	88 (29.83)	42 (25.77)	46 (34.85)
>50	33 (9.59)	21 (11.05)	12 (7.79)	13 (4.41)	9 (5.52)	4 (3.03)
**Epithelium**						
0	180 (90.00)	98 (90.74)	82 (89.13)	0	0	0
>0 < 1	12 (6.00)	9 (8.33)	3 (3.26)	2 (0.74)	1 (0.68)	1 (0.82)
≥1–≤10	6 (3.00)	1 (0.93)	5 (5.43)	71 (26.39)	41 (27.89)	30 (24.59)
>10–≤50	2 (1.00)	0	2 (2.17)	166 (61.71)	86 (58.50)	80 (65.57)
>50	0	0	0	30 (11.15)	19 (12.93)	11 (9.02)
**Binary, with 1% cut-off**						
**Stroma**						
<1	167 (48.55)	98 (51.58)	69 (44.81)	51 (17.29)	30 (17.40)	21 (15.91)
≥1	177 (51.45)	92 (48.42)	85 (55.19)	244 (82.71)	133 (81.60)	111 (84.09)
Epithelium						
<1	192 (96.00)	107 (99.07)	85 (92.39)	2 (0.74)	1 (0.68)	1 (0.82)
≥1	8 (4.00)	1 (0.93)	7 (7.61)	267 (99.26)	146 (99.32)	121 (99.18)
**Binary, with 10% cut-off**						
**Stroma**						
≤10	247 (71.80)	135 (71.05)	112 (72.73)	194 (65.76)	112 (68.71)	82 (62.12)
>10	97 (28.20)	55 (28.95)	42 (27.27)	101 (34.24)	51 (31.29)	50 (37.88)
Epithelium						
≤10	198 (99.00)	108 (100.00)	90 (97.83)	73 (27.14)	42 (28.57)	31 (25.41)
>10	2 (1.00)	0	2 (2.17)	196 (72.86)	105 (71.43)	91 (74.59)

TDLU, terminal duct lobular unit.

For each woman, TMA included 3 cores from normal TDLUs; the number of benign lesion cores per woman ranged between 3 and 6 (median 3). We found strong correlation in CD44 expression across available cores for a woman both for normal cores (ICC = 0.80, 95% CI 0.72, 0.87 for stroma; ICC = 0.76, 95% CI 0.66, 0.84 for epithelium; ICC = 0.71, 95% CI 0.60, 0.80 for combined epithelium/stroma) and lesions (ICC = 0.74, 95% CI 0.63, 0.82 for stroma; ICC = 0.78, 95% CI 0.68, 0.85 for epithelium; ICC = 0.58, 95% CI 0.44–0.70 for combined epithelium/stroma) (Table 3). Strong correlations in CD24 expression were observed in normal cores for stroma (ICC = 0.63, 95% CI 0.50, 0.74) and moderate correlations were observed in normal cores for epithelium (ICC = 0.45, 95% CI 0.28; 0.59) and combined epithelium/stroma (ICC = 0.48, 95% CI 0.32; 0.62) as well as for all tissue types in lesions (ICC = 0.45, 95% CI 0.30; 0.59 for stroma; ICC = 0.42, 95% CI 0.25; 0.57 for epithelium; and ICC = 0.49, 95% CI 0.34; 0.63 for epithelium/stroma). Moderate correlations in ALDH1A1 expression were observed for all tissue types in both TDLU cores (ICC range 0.51–0.58) and benign lesions (ICC range 0.44–0.54).

**TABLE 3:** Intra-class correlation in marker expression across available cores for a woman, by core and tissue type as assessed by Definiens.

Tissue type	Core type	
	Normal tissue	Benign lesions
**CD44**		
Stroma	0.80 (0.72, 0.87)	0.74 (0.63, 0.82)
Epithelium	0.76 (0.66, 0.84)	0.78 (0.68, 0.85)
Combined epi + stroma	0.71 (0.60, 0.80)	0.58 (0.44, 0.70)
**CD24**		
Stroma	0.63 (0.50, 0.74)	0.45 (0.30, 0.59)
Epithelium	0.45 (0.28, 0.59)	0.42 (0.25, 0.57)
Combined epi + stroma	0.48 (0.32, 0.62)	0.49 (0.34, 0.63)
**ALDH1A1**		
Stroma	0.51 (0.35, 0.64)	0.54 (0.39, 0.67)
Epithelium	0.55 (0.40, 0.68)	0.44 (0.27, 0.59)
Combined epi + stroma	0.58 (0.45, 0.70)	0.45 (0.29, 0.59)

### 3.2 Agreement between pathologist and Definiens readings

**FIGURE 3:**
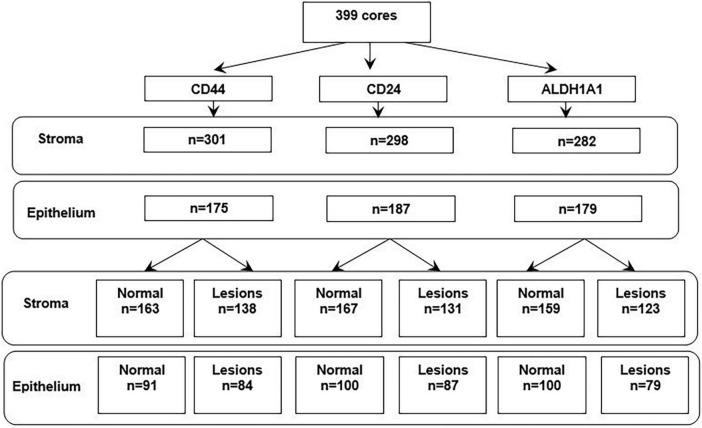
The number of cores in the study with available readings from pathologist and Definiens, by tissue type.

**FIGURE 4:**
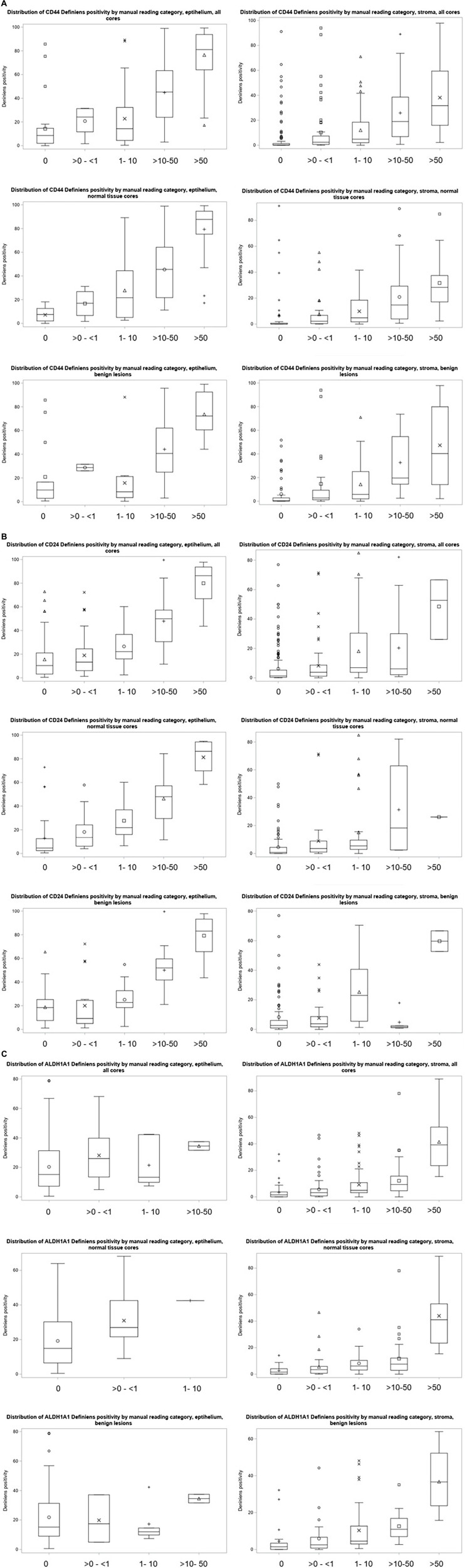
**(A)** Distribution of CD44 Definiens positivity according to the manual reading categories. **(B)** Distribution of CD24 Definiens positivity according to the manual reading categories. **(C)** Distribution of ALDH1A1 Definiens positivity according to the manual reading categories.

Of 399 cores in this study, the pathologist and Definiens readings were available for 301 cores for CD44 (163 normal tissue and 138 for lesions), 298 cores for CD24 (167 for normal and 131 for lesions), and 282 cores for ALDH1A1 (159 for normal and 123 for lesions) for stroma and 175 cores for CD44 (91 for normal and 84 for lesions), 187 cores for CD24 (100 for normal and 87 for lesions), and 179 cores for ALDH1A1 (100 for normal and 79 for lesions) for epithelium (Figure 3). Distribution of continuous Definiens readings by the category of manual assessment is presented in Figure 4.

Overall percent agreement between manual and Definiens scoring was 42% for stroma and 49% for epithelium for CD44, 24% for stroma and 26% for epithelium for CD24, and 40% for stroma and 33% for epithelium for ALDH1A1. For stroma, we found a strong correlation between pathologist and Definiens readings for CD44 (correlation coefficient *r* = 0.64, 95% CI 0.53; 0.72 for normal TDLU cores and *r* = 0.60, 95% CI 0.48; 0.70 for benign lesions) and normal TDLU cores for ALDH1A1 (*r* = 0.60, 95% CI 0.49; 0.69). We found moderate correlations for normal TDLU cores for CD24 (*r* = 0.40, 95% CI 0.26; 0.52) and for benign lesion cores for ALDHA1A1 (*r* = 0.57, 95% CI 0.44; 0.68) (Table 4).

**TABLE 4:** Correlation of stem cell marker expression from pathologist and Definiens assessments [Spearman rank correlation coefficient (95% CI and *p*-value) for categorical; sensitivity (95% CI) and specificity (95% CI) for binary variables].

Tissue type	CD44				CD24				ALDH1A1			
	*N*	Normal TDLUs	*N*	Benign lesions	*N*	Normal TDLUs	*N*	Benign lesions	*N*	Normal TDLUs	*N*	Benign lesions
***Categorical (5 levels)***												
Stroma	163	0.64 (0.53; 072) (<0.001)	138	0.60 (0.48; 0.70) (<0.001)	167	0.40 (0.26; 0.52) (<0.0001)	131	0.24 (0.07; 0.39) (0.01)	159	0.60 (0.49; 0.69) (<0.001)	123	0.57 (0.44; 0.68) (< 0.001)
Epithelium	91	0.68 (0.55; 0.78) (<0.001)	84	0.61 (0.46; 0.73) (<0.001)	100	0.66 (0.53; 0.76) (<0.001)	87	0.64 (0.49; 0.75) (<0.001)	100	0.14 (–0.06; 0.33) (0.16)	79	0.01 (–0.22; 0.23) (0.96)
***Binary, 1% as cut-off***												
**Stroma**												
Sensitivity	163	0.95 (0.90; 1.00)	138	0.91 (0.83; 0.98)	167	0.92 (0.83; 1.00)	131	0.95 (0.85; 1.00)	159	0.93 (0.88; 0.98)	123	0.97 (0.94; 1.00)
Specificity	163	0.60 (0.50; 0.71)	138	0.50 (0.39; 0.61)	167	0.48 (0.40; 0.57)	131	0.24 (0.16; 0.32)	159	0.31 (0.21; 0.42)	123	0.36 (0.23; 0.49)
**Epithelium**												
Sensitivity	91	1.00	84	0.97 (0.93; 1.00)	100	1.00	87	1.0	100	1.00	79	1.00
Specificity	91	0.18 (0.00; 0.36)	84	0.06 (0.00; 0.18)	100	0.04 (0.00; 0.09)	87	0	100	0.01 (0.00; 0.03)	79	0.01 (0.00; 0.04)
***Binary, 10% as cut-off***												
**Stroma**												
Sensitivity	163	0.67 (0.54; 0.81)	138	0.88 (0.77; 0.99)	167	0.63 (0.29; 0.96)	131	0.43 (0.06; 0.80)	159	0.64 (0.51; 0.77)	123	0.70 (0.56; 0.85)
Specificity	163	0.80 (0.73; 0.88)	138	0.76 (0.68; 0.84)	167	0.82 (0.76; 0.88)	131	0.73 (0.66; 0.81)	159	0.85 (0.78; 0.92)	123	0.79 (0.70; 0.88)
**Epithelium**												
Sensitivity	91	1.00	84	0.95 (0.89; 1.00)	100	1.00	87	1.00	100	NA	79	1.00
Specificity	91	0.46 (0.29; 0.62)	84	0.52 (0.34; 0.70)	100	0.41 (0.29; 0.53)	87	0.35 (0.22; 0.48)	100	NA	79	0.35 (0.24; 0.46)

NA, not available; TDLU, terminal duct lobular unit.

For epithelium, strong correlations were observed for marker expression readings for CD44 for normal TDLU cores (*r* = 0.68, 95% CI 0.55; 0.78) and benign lesions (*r* = 0.61, 95% CI 0.46; 073) and for CD24 for normal cores (*r* = 0.66, 95% CI 0.53; 0.76) and benign lesions (*r* = 0.64, 95% CI 0.49; 0.75); no correlations were found for ALDH1A1.

In a secondary analyses, we also examined correlations between pathologist and Definiens assessments after combining the lowest 2 categories (0 and <1%) and then the lowest 3 categories (0, <1, and 1–10%) (Supplementary Table 1). Overall, there were no appreciable differences in the strength of the correlations with these approaches. Correlation coefficients were slightly attenuated for CD44 for stroma in all cores, normal tissue cores, and benign lesions and improved for ALDH1A1 for stroma. Remaining estimates fluctuated slightly but remained very similar to the original results.

With 1% positivity cut-off for stroma, sensitivity ranged between 0.92–0.95 normal TDLU cores and 0.91–0.97 for benign lesions and specificity ranged between 0.31–0.60 for normal TDLU cores and 0.24–0.50 for being lesions. With 10% cut-off for stroma, sensitivity ranged between 0.63–0.67 for normal TDLU cores and 0.43–0.88 for benign lesions and specificity ranged between 0.80–0.85 for normal cores and 0.73–0.79 for benign lesions. For epithelium, 10% cut-off for all markers resulted in better estimates for both sensitivity and specificity in normal TDLU cores (except for ALDH1A1) and benign lesions (Table 4). In additional analyses accounting for correlation across available cores for a woman, the results for correlations as well as sensitivity and specificity remained similar (Supplementary Table 2).

## 4 Discussion

In this study comparing manual pathologist and computer-automated assessments of CD44, CD24, and ALDH1A1 expression in 399 cores from 81 cancer-free women with benign breast biopsies, we found wide variation in expression of all three markers in both stroma and epithelium in normal tissue cores and benign lesions. We observed moderate to strong correlations between marker expressions across available cores for a woman for all three markers and, for the most part, moderate to strong correlations between assessment of immunoreactivity by pathologist and Definiens.

Recently, the role of stem cells in breast carcinogenesis was recognized as a high priority for translational research (4). However, the data on the expression of stem cell markers CD44, CD24, and ALDH1A1 in benign breast tissue of cancer-free women remains extremely limited. The progress in this area is further hindered by the absence of established cut-offs for assessment of positivity in benign as well as tumor tissue with various studies in tumors using different cut points (33–38). In addition, manual assessment of immunoreactivity is subjective, semi-quantitative, incorporating both intensity and extent of immunoreactivity, and is expensive, time-consuming, reliant on subjective scoring parameters, and potentially prone to bias as the pathologist cannot be blinded to the histology of the evaluated tissue sample. Automated image analysis of TMAs offers unprecedented opportunity for studying various tissue markers, including stem cell markers, in large-scale population-based studies by providing objective, reliable, and faster assessment of IHC results while minimizing pathologist involvement. In this study, we aimed not only to examine the expression of CD44, CD24, and ALDH1A1 in various tissue types and cores but also to assess the agreement between their expression assessed by a pathologist and by using computerized deep-machine learning approaches.

Our findings suggest (1) a strong correlation in CD44 expression in stroma and in epithelium across available cores for a woman both for normal TDLU cores and benign lesions, (2) a strong correlation in CD24 expression in stroma in normal TDLU cores and moderate correlations in lesions as well as epithelium in both normal TDLU and benign lesion cores, and (3) moderate correlations in ALDH1A1 expression in stroma and in epithelium in normal TDLU cores and benign lesions. Our data demonstrate that despite variability in the markers, the correlation across the cores is reasonably high. Though no previous studies examined these correlations in cancer-free women, in an earlier study using breast tumor TMAs from Mayo Clinic, we also found strong correlation across available cores (2–3 for a woman) for all three markers (ICC = 0.82, 0.78, and 0.58 for CD44, CD24, and ALDH1A1, respectively), though the findings were not examined separately by the tissue type and the expression assessment was based on pathologist assessment only (29).

Overall, we found moderate to strong correlation between pathologist and Definiens assessment. Previous studies have generally demonstrated good agreement between pathologist- and computationally generated scores for a few tumor markers (39–44). In a recent investigation of 17 tissue markers (AR, CD20, CD4, CD8, CD163, EPRS, ER, FASN, H3K27, IGF1R, IR, Ki67, phospho-mTOR, PR, PTEN, RXR, and VDR) in breast tumor tissue from 5,914 participants in NHS and NHSII, the correlations between pathologist and Definiens assessments ranged from weak to strong and were ≥0.55 for 10 of the 17 markers analyzed (44). Similar strong correlations were observed by other research groups that used AQUA algorithms and automated algorithms designed with MatLab for evaluation of some of these markers (39, 40, 45, 46). Further, strong correlations were also found in studies that utilized Definiens approach in other types of tumor tissue (prostate and esophageal cancers) (47, 48). However, whether this agreement extends to biomarkers in normal breast tissue and benign lesions is poorly understood. Our findings in normal breast tissue and benign lesions are in line with the previous reports on other markers, though, to our knowledge, stem cell markers have not been investigated in this capacity in any prior studies.

We next examined the cores that had discordant readings from pathologist and Definiens to identify potential reasons for discrepancies for each for the markers. Definiens readings appeared to be greater than manual assessment results for the majority of these discrepancies. In most of these cases, however, continuous Definiens scores were closer to the lower end of the manually assigned category. In general, we identified three possible explanation for discrepancies: (1) tissue folding; (2) tissue segmentation misclassification–stromal positivity surrounding epithelial cells being picked up as epithelial staining or stromal cells close to epithelium being segmented as epithelium (interstitial stromal cells); and (3) ability of the Definiens to pick up lower-intensity staining signals not captured by human eye (Supplementary Figures 1A–C).

Only a small proportion of cores were assigned a lower category by Definiens as compared to manual reading. Some of these discrepancies represented true false-negative results, while a few were the result of tissue folding as well as some of the stromal cells being were captured by Definiens as epithelium thus lowering the staining score for stroma. Finally, as Definiens picks up more cells (cellularity) than might be counted by the naked eye, the staining read percentage could be reduced even when the number of positively stained cells is the same from manual and Definiens assessment.

Our study is the largest to date to describe distribution of CD44, CD24, an ALDH1A1 in normal tissue and benign lesions from cancer-free women (including their heterogeneity across the cores for a woman), and to investigate, for the first time, the agreement between manual assessment and that from automated image analysis with Definiens. The study used data and samples from the Nurses’ Health Study and Nurses’ Health Study II, established cohorts with more than 30 years of follow-up, confirmed benign breast disease status, and comprehensive information on breast cancer risk factors. Use of data collected within a population-based cohort allowed us to leverage real-world data collected across different pathology departments, with varying tissue processing protocols, making our results more generalizable to a wider group of researchers. In our study, we compared a semi-automated computational platform to pathologist assessments. All manual readings were performed by a single expert pathologist. Definiens software only allows a maximum of 12 cores to be included for algorithm training. This is a limitation of the software as 12 cores may be insufficient to adequately train tissue segmentation. Importantly, in our recent study we demonstrated that IHC staining quantification is highly comparable across various software applications for IHC analysis [Definiens, InForm^®^ (Akoya Biosciences), and QuPath] (49).

As storage time can potentially affect the marker expression levels (50), we examined the expression of each marker in newer (biopsy date up to 1981) versus older (biopsy date after 1981) samples. For all three markers, the expression in stroma and epithelium appeared to be slightly higher in newer samples. While the expression of stem cell markers may be underestimated in older samples, importantly, our findings on expression patterns across tissue (stroma, epithelium) and core types (lesions, normal TDLUs) remained unchanged regardless of the sample years. Further, as our comparison of pathologist vs. Definiens readings was done on core-by-core basis, the year of sample collection would not affect our findings. Similarly, as testing homogeneity across the cores is based on the cores from the same biopsy sample (i.e., biopsy year), these finding also would not be affected by the biopsy year.

In conclusion, we described expression of CD44, CD24, and ALDH1A1 in benign biopsy tissue and compared assessment of immunoreactivity by pathologist and computational approach. Our data indicate that, despite sufficient variability in markers’ expression, the correlation across the cores for a woman is reasonably high. Our findings show that Definiens semi-automated digital image analysis system can provide results comparable to those obtained by an expert pathologist for these markers and that cut points for computationally derived data may require marker-specific optimization. Importantly, pilot studies may be needed before any large-scale investigations to improve agreement with pathologist’s evaluations prior to wider study implementation.

## Data availability statement

The datasets presented in this article are not readily available because data are available upon reasonable written request. According to standard controlled access procedure, applications to use NHS/NHSII/HPFS resources will be reviewed by our External Collaborators Committee for scientific aims, evaluation of the fit of the data for the proposed methodology, and verification that the proposed use meets the guidelines of the Ethics and Governance Framework and the consent that was provided by the participants. Investigators wishing to use NHS/NHSII/HPFS data are asked to submit a brief description of the proposed project [go to https://www.nurseshealthstudy.org/researchers (contact email: nhsaccess@channing.harvard.edu) and https://sites.sph.harvard.edu/hpfs/for-collaborators/ for details]. Requests to access the datasets should be directed to nhsaccess@channing.harvard.edu.

## Ethics statement

The studies involving human participants were reviewed and approved by Institutional Review Boards of the Brigham and Women’s Hospital and Harvard T.H. Chan School of Public Health and those of participating registries. The patients/participants provided their written informed consent to participate in this study.

## Author contributions

LY and RT: conceptualization and writing—original draft. MM, GB, VB-M, RT, DM, and YH: data curation. LY, YM, and BR: formal analysis. LY, RT, YH, and GB: methodology. LY: supervision. All authors contributed to writing—review and editing, contributed to the article, and approved the submitted version.
