# Supplementary material for: Reliability of CD44, CD24, and ALDH1A1 immunohistochemical staining: Pathologist assessment compared to quantitative image analysis
Source: Front Med (Lausanne). 2022 Dec 14;9:1040061. doi: 10.3389/fmed.2022.1040061 (PMC9794585; doi:10.3389/fmed.2022.1040061)
Supplement: Supplementary file 1 [file Data_Sheet_1.doc]

**Supplementary Table 1. Correlations between pathologist reading and Definiens assessment with combined categories**

| **Tissue type** | **CD44** | | | | **CD24** | | | | | **ALDH1A1** | | | |
| --- | --- | --- | --- | --- | --- | --- | --- | --- | --- | --- | --- | --- | --- |
|  | **N** | **Normal TDLUs** | **N** | **Benign lesions** | **N** | **Normal TDLUs** | **N** | **Benign lesions** | **N** | | **Normal TDLUs** | **N** | **Benign lesions** |
| ***Categorical (***<1, 1- 10, >10-50, and >50%***)*** |  |  |  |  |  |  |  |  |  | |  |  |  |
| Stroma | 163 | 0.60 (0.49; 0.69)  (<0.0001) | 138 | 0.58 (0.46; 0.68) (<0.0001) | 167 | 0.35 (0.21; 0.48) (<0.0001) | 131 | 0.26 (0.09; 0.41) (0.003) | 159 | | 0.59 (0.48; 0.69) (<0.0001) | 123 | 0.57 (0.43; 0.68)  (<0.0001) |
| Epithelium | 91 | 0.68 (0.55; 0.77) (<0.0001) | 84 | 0.61 (0.45; 0.73) (<0.0001) | 100 | 0.65 (0.52; 0.75) (<0.0001) | 87 | 0.67 (0.54; 0.77) (<0.0001) | 100 | | 0.07 (-0.13; 0.26) (0.56) | 79 | 0.01 (-0.21; 0.23)  (0.91) |
| ***Categorical (***0 - 10, >10-50, and >50%***)*** |  |  |  |  |  |  |  |  |  | |  |  |  |
| Stroma | 163 | 0.48 (0.35; 0.59) (<0.0001) | 138 | 0.58 (0.46; 0.68) (<0.0001) | 167 | 0.25 (0.10; 0.39) (0.001) | 131 | 0.12 (-0.05; 0.29) (0.17) | 159 | | 0.59 (0.48; 0.68) (<0.0001) | 123 | 0.52 (0.38; 0.64)  (<0.0001) |
| Epithelium | 91 | 0.65 (0.51; 0.75) (<0.0001) | 84 | 0.63 (0.49; 0.75) (<0.0001) | 100 | 0.62 (0.49; 0.73) (<0.0001) | 87 | 0.69 (0.56; 0.78) (<0.0001) | 100 | | NA | 79 | 0.09 (-0.13; 0.30)  (0.43) |

Abbreviations: TDLU – terminal duct lobular unit

**Supplementary table 2. Correlation of stem cell marker expression from Pathologist and Definiens assessments, accounting for correlation across available cores for a woman (Spearman rank correlation coefficient [95% CI and p-value] for categorical; sensitivity [95% CI] and specificity [95% CI] for binary variables)**

| **Tissue type** | **CD44** | | | | **CD24** | | | | **ALDH1A1** | | | |
| --- | --- | --- | --- | --- | --- | --- | --- | --- | --- | --- | --- | --- |
|  | **N** | **Normal TDLUs** | **N** | **Benign lesions** | **N** | **Normal TDLUs** | **N** | **Benign lesions** | **N** | **Normal TDLUs** | **N** | **Benign lesions** |
| ***Categorical (5 levels)*** |  |  |  |  |  |  |  |  |  |  |  |  |
| Stroma | 163 | 0.61 (0.41; 0.75) (<0.001) | 138 | 0.52 (0.25; 0.71) (<0.001) | 167 | 0.42 ( (0.25,0.56) (<0.001) | 131 | 0.27 (-0.03; 0.52) (0.07) | 159 | 0.59 (0.43; 0.72) (<0.001) | 123 | 0.58 (0.32; 0.75)  (<0.001) |
| Epithelium | 91 | 0.57 (0.32; 0.75) (<0.001) | 84 | 0.59 (0.35; 0.75) (<0.001) | 100 | 0.61 (0.39; 0.77) (<0.001) | 87 | 0.59 (0.38; 0.75) (<0.001) | 100 | 0.12 (-0.17; 0.39) (0.42) | 79 | -0.10 (-0.34; 0.16)  (0.46) |
| ***Binary, 1%as cut-off*** |  |  |  |  |  |  |  |  |  |  |  |  |
| Stroma |  |  |  |  |  |  |  |  |  |  |  |  |
| Sensitivity | 163 | 0.95 (0.87; 0.98) | 138 | 0.91 (0.79; 0.96) | 167 | 0.92 (0.78; 0.97) | 131 | 0.95 (0.70; 0.99) | 159 | 0.93 (0.86; 0.97) | 123 | 0.97 (0.90; 0.99) |
| Specificity | 163 | 0.60 (0.45; 0.74) | 138 | 0.50 (0.36; 0.64) | 167 | 0.48 (0.38; 0.60) | 131 | 0.24 (0.17; 0.33) | 159 | 0.31 (0.21; 0.45) | 123 | 0.36 (0.23; 0.52) |
| Epithelium |  |  |  |  |  |  |  |  |  |  |  |  |
| Sensitivity | 91 | 0.99 (0.95; 1.00) | 84 | 0.97 (0.89; 0.99) | 100 | 1.0 | 87 | 1.0 | 100 | 1.0 | 79 | 1.0 |
| Specificity | 91 | 0.12 (0.04; 0.31) | 84 | 0.06 (0.01; 0.29) | 100 | 0.04 (0.01; 0.23) | 87 | 0 | 100 | 0.01 (0.00; 0.07) | 79 | 0.01 (0.00; 0.09) |
| ***Binary, as 10% cut-off*** |  |  |  |  |  |  |  |  |  |  |  |  |
| Stroma |  |  |  |  |  |  |  |  |  |  |  |  |
| Sensitivity | 163 | 0.67 (0.49; 0.82) | 138 | 0.88 (0.70; 0.96) | 167 | 0.63 (0.26; 0.89) | 131 | 0.43 (0.10; 0.83) | 159 | 0.64 (0.50; 0.76) | 123 | 0.70 (0.49; 0.85) |
| Specificity | 163 | 0.80 (0.69; 0.88) | 138 | 0.76 (0.64; 0.85) | 167 | 0.82 (0.74; 0.89) | 131 | 0.73 (0.62; 0.83) | 159 | 0.85 (0.75; 0.92) | 123 | 0.79 (0.69; 0.86) |
| Epithelium |  |  |  |  |  |  |  |  |  |  |  |  |
| Sensitivity | 91 | 0.97 (0.92; 0.99) | 84 | 0.95 (0.85; 0.98) | 100 | 1.0 | 87 | 1.0 | 100 | Non-estimable | 79 | 1.0 |
| Specificity | 91 | 0.48 (0.33; 0.64) | 84 | 0.52 (0.34; 0.69) | 100 | 0.41 (0.28; 0.56) | 87 | 0.35 (0.22; 0.51) | 100 | 0.39 (0.26; 0.53) | 79 | 0.35 (0.26; 0.46) |

**Abbreviations:** TDLU – terminal duct lobular unit
