# Supplementary figures and images for: Reliability of CD44, CD24, and ALDH1A1 immunohistochemical staining: Pathologist assessment compared to quantitative image analysis
Source: Front Med (Lausanne). 2022 Dec 14;9:1040061. doi: 10.3389/fmed.2022.1040061 (PMC9794585; doi:10.3389/fmed.2022.1040061)

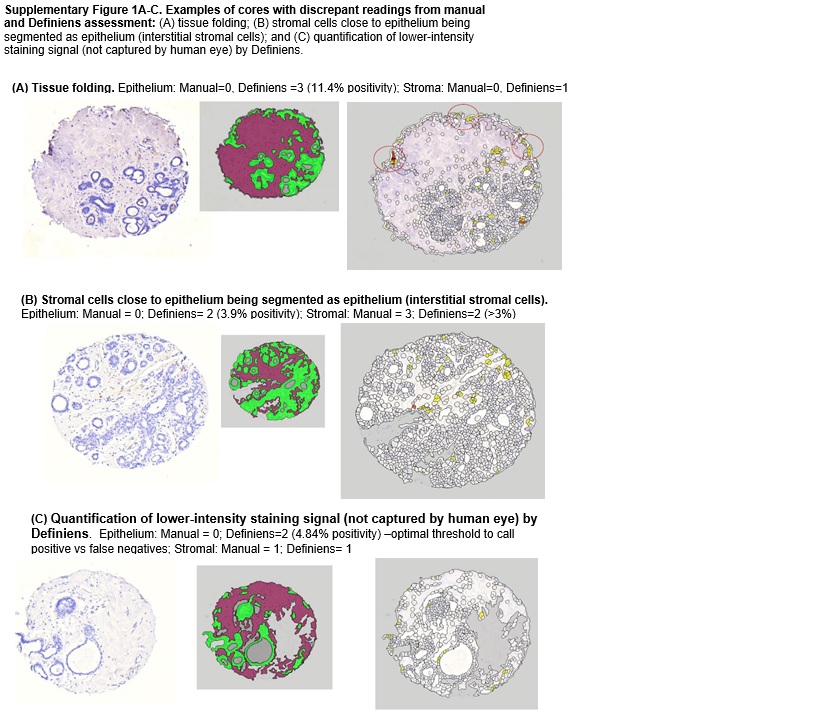

Supplement: Supplementary file 2 [file Image_1.JPEG]
